# When Even a Robot Tutor Zooms: A Study of Embodiment, Attitudes, and Impressions

**DOI:** 10.3389/frobt.2021.679893

**Published:** 2021-06-30

**Authors:** Junko Kanero, Elif Tutku Tunalı, Cansu Oranç, Tilbe Göksun, Aylin C. Küntay

**Affiliations:** ^1^ Faculty of Arts and Social Sciences, Sabancı University, Istanbul, Turkey; ^2^ MPRG iSearch, Max Planck Institute for Human Development, Berlin, Germany; ^3^ Department of Psychology, Koç University, Istanbul, Turkey

**Keywords:** human-robot interaction, second language learning (L2 learning), embodiment, attitudes, impressions

## Abstract

This study used an online second language (L2) vocabulary lesson to evaluate whether the physical body (i.e., embodiment) of a robot tutor has an impact on how the learner learns from the robot. In addition, we tested how individual differences in attitudes toward robots, first impressions of the robot, anxiety in learning L2, and personality traits may be related to L2 vocabulary learning. One hundred Turkish-speaking young adults were taught eight English words in a one-on-one Zoom session either with a NAO robot tutor (*N* = 50) or with a voice-only tutor (*N* = 50). The findings showed that participants learned the vocabulary equally well from the robot and voice tutors, indicating that the physical embodiment of the robot did not change learning gains in a short vocabulary lesson. Further, negative attitudes toward robots had negative effects on learning for participants in the robot tutor condition, but first impressions did not predict vocabulary learning in either of the two conditions. L2 anxiety, on the other hand, negatively predicted learning outcomes in both conditions. We also report that attitudes toward robots and the impressions of the robot tutor remained unchanged before and after the lesson. As one of the first to examine the effectiveness of robots as an online lecturer, this study presents an example of comparable learning outcomes regardless of physical embodiment.

## Introduction

*Social robots*, robots that interact and communicate with humans by following the behavioral norms of human-human interactions (e.g., Bartneck and Forlizzi, 2004; Kanero et al., 2018), are becoming abundant across a variety of settings such as homes, hospitals, and schools. A particularly interesting application of social robots is language education because of the significance of the topic as well as the unique characteristics of social robots. Language education is critical for people of all ages. For children, language abilities are known to predict future academic achievement and social skills (Hoff, 2013; Milligan et al. 2007); for adults, language skills can broaden social and occupational opportunities (e.g., Paolo and Tansel, 2015). Learning another language can also contribute to the development of cognitive skills in children (Kovács and Mehler, 2009), and the attainment of them in older adults (Bialystok et al., 2004). Importantly, a wealth of research in psychology and education suggests that learning both first (L1) and second language (L2) requires *interactions* (Verga and Kotz, 2013; Konishi et al., 2014; Lytle and Kuhl, 2017). As a social agent with a physical body, a robot can play the role of a tutor through vocal, gestural, and facial expressions to provide an interactive learning experience (Han et al., 2008; Kennedy et al., 2015; Kanero et al., 2018). The current study focuses on *embodiment* and examines whether and how important it is for L2 learners to interact with a robot tutor with a physical body.

The general bodily affordances of social robots were suggested to improve the learning experience as they can engage with the learners’ physical world and elicit social behaviors from them (Belpaeme et al., 2018). For instance, when teaching a new word, robots can perform gestures with their hands to depict the target object or direct the learner’s attention to the object with their eyes, both of which are an integral part of interacting and learning with robots (Admoni and Scassellati, 2017; Kanero et al., 2018). Some studies indicate that interacting with a robot in person or through a screen may not have much of a difference in terms of learning (e.g., Kennedy et al., 2015), and studies on language learning with intelligent virtual agents provide support to this (Macedonia et al., 2014). In fact, a study on second language learning found participants performing worse after interacting with a physically present robot as opposed to its virtual version or a voice-only tutor, speculatively because it was too novel and interesting, hence distracting, for the participants (Rosenthal-von der Pütten et al., 2016). On the other hand, there is also research suggesting that interacting with a physically present robot may yield better outcomes. For instance, one study found that adults performed better in solving logic puzzles when they were partnered off with a physically present robot as opposed to a disembodied voice or a video of a robot (Leyzberg et al., 2012), though solving a logic puzzle is inherently different from learning a language.

*Embodiment* has been defined in many different ways partially because the term is used in various disciplines including philosophy, psychology, computer science, and robotics (see Deng et al., 2019). One of the clear definitions provided by roboticists is that of Pfeifer and Scheier (1999): “In artificial systems, the term refers to the fact that a particular agent is realized as a physical robot or as a simulated agent” (p. 649). Focusing on the social aspect, Barsalou et al. (2003) states that embodiment is the “states of the body, such as postures, arm movements, and facial expressions, [which] arise during social interaction and play central roles in social information processing” (p. 43). In human-robot interaction, Li (2015) made a distinction between what he calls *physical presence* and *physical embodiment* to systematically evaluate the different bodily affordances of robots. According to Li (2015), *physical presence* differentiates a robot in the same room with the user and a robot appearing on the screen. On the other hand, *physical embodiment* differentiates a (co-present or telepresent) materialized robot and a virtual agent (e.g., a computer-generated image of a robot).

The review by Li (2015) concluded that the physical presence of the robot, but not its embodiment, has a positive influence on social interactions. Critically, however, the conclusion was drawn based on four studies from three publications only. Overall, while previous research provides valuable insights into how different dimensions of physicality influence human-robot interaction, they fall short in revealing the difference between having and not having a body and face on learning outcomes. Although their appearance can simulate different animate agents such as a human or an animal, all social robots have a body and face. How does this influence people’s learning, as opposed to not having either? Following the distinctions drawn by Li (2015), we compare a robot tutor (embodied but not physically present) with a voice-only tutor (not embodied nor physically present) in an online lesson to understand the effects of physical embodiment.

Research also suggests that embodiment may have different implications for different people, as in individuals with Autism Spectrum Disorder struggling with understanding the emotions of a virtual agent than a real agent, whether it is a robot or a human, in contrast to typically developed individuals (Chevalier et al., 2017). People’s varying attitudes toward robots may also influence their preference for a physical or virtual robot (Ligthart and Truong, 2015). Another study with children also suggests that age and experience may diminish the effect of physical presence, as it found that younger children with hearing impairments learned more words in sign language when they interacted with a physically present robot than a video of it, whereas older children with more experience in sign language equally benefited from both (Köse et al., 2015). Therefore, the current study further explores interrelations among individual differences (specifically attitudes toward robots, first impressions of the robot tutor, anxiety about learning a second language, and personality traits) and learning outcomes across different degrees of embodiment.

Although not much is known specifically about the effects of individual differences in learning with robots, some studies have explored how attitudes and personality are related to the ways in which a person interacts with a robot. For example, the patterns of speech and eye gaze were observed while adults built an object with a humanoid robot (Ivaldi et al., 2017). The study found that individuals with negative attitudes toward robots tended to look less at the robot’s face and more at the robot’s hands. In another study, when approached by a robot, individuals with high levels of negative attitudes toward robots and the personality trait of neuroticism kept a larger personal space between the robot and themselves (Takayama and Pantofaru, 2009).

In the case of language learning, Kanero et al. (2021) were first to examine how attitudes toward robots, anxiety about learning L2, and personality may predict the learning outcomes of a robot-led L2 vocabulary lesson. The study found that negative attitudes measured through the Negative Attitudes toward Robots Scale (NARS; Nomura et al., 2006) as well as anxiety about learning L2 measured through the Foreign Language Classroom Anxiety Scale (FLCAS; Horwitz et al., 1986) predicted the number of words participants learned in an in-person vocabulary lesson with a robot tutor. The results also showed that the robot was an effective language tutor, akin to a human tutor. However, it is unclear whether the tutor robot is as effective when it is not physically present, and whether individual differences such as attitudes toward robots and L2 anxiety predict the learning outcomes for a telepresent robot tutor.

In addition to the individual difference measures used in the previous study (Kanero et al., 2021), the current study also assesses the learners’ impressions of robots, which are expected to affect their engagement in the long run. Previous studies in human-human interaction suggest that the first impression is formed very quickly after just seeing a picture of an individual and might remain unchanged even after meeting and interacting with the same individual in person (e.g., Gunaydin et al., 2017; see also Willis and Todorov, 2006). However, it is unclear whether the same principle applies to commercial social robots (e.g., NAO), which are inanimate objects with a homogeneous appearance shared across individuals. Therefore, in the current study, we included an additional measure to examine if the impressions of the robot have a role in robot-led learning. Further, we evaluate whether the impressions of the robot tutor as well as attitudes toward robots change before and after interacting with the robot tutor.

In summary, this study explores the impact of having a body in robot-led language lessons by comparing a robot tutor and a voice-only tutor in terms of learning outcomes as well as the influence of attitudes, impressions, L2 anxiety, personality. We also report the details of the learner’s general attitudes toward roborts, impressions of the robot tutor, and preferences to the specific type of tutor (robot vs. voice vs. human). In the Discussion, we also compare our data to the data of the previous study (Kanero et al., 2021) to address whether the physical presence in robot-led language lessons would affect these factors.

## Methods

### Participants

The dataset consisted of 100 native Turkish-speaking young adults: 50 in the robot tutor condition (*age range* = 18–32 years; *M*_*ag*e_ = 23.49 years; *SD* = 2.53; 33 females, 17 males), and 50 in the voice tutor condition (*age range* = 18–35 years; *M*_*age*_ = 24.15 years; *SD* = 3.62; 33 females, 16 males, 1 other). We relied on a convenience sample, and participants were recruited through advertisements on social media as well as word of mouth. Before the lesson, the average English test score of participants (Quick Placement Test; University of Cambridge Local Examinations Syndicate [UCLES], 2001) was 39.68 out of 60 in the robot tutor condition (*score range* = 16–58; *SD* = 9.07) and 37.64 in the voice tutor condition (*score range* = 20–55; *SD* = 9.25). Participants had no known vision or hearing impairments. One participant in the robot tutor condition did not show up for the second session, and thus the two delayed language tests and the post-lesson survey were not administered to this participant. In addition, one participant in the robot tutor condition was not taught one of the eight vocabulary words due to a technical error, and thus the test data for that word were not used. Participants were given a gift card for their participation.

### Materials and Procedures

**FIGURE 1:**
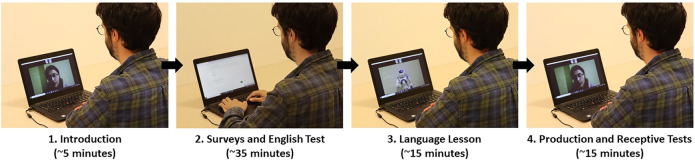
The procedure of the lesson from the participant’s perspective. In the voice-only tutor condition, the voice sound spectrum appeared instead of the robot (see Figure 2 and Supplementary Material). Step 4 (Production and Receptive Tests) was repeated one week later.

**FIGURE 2:**
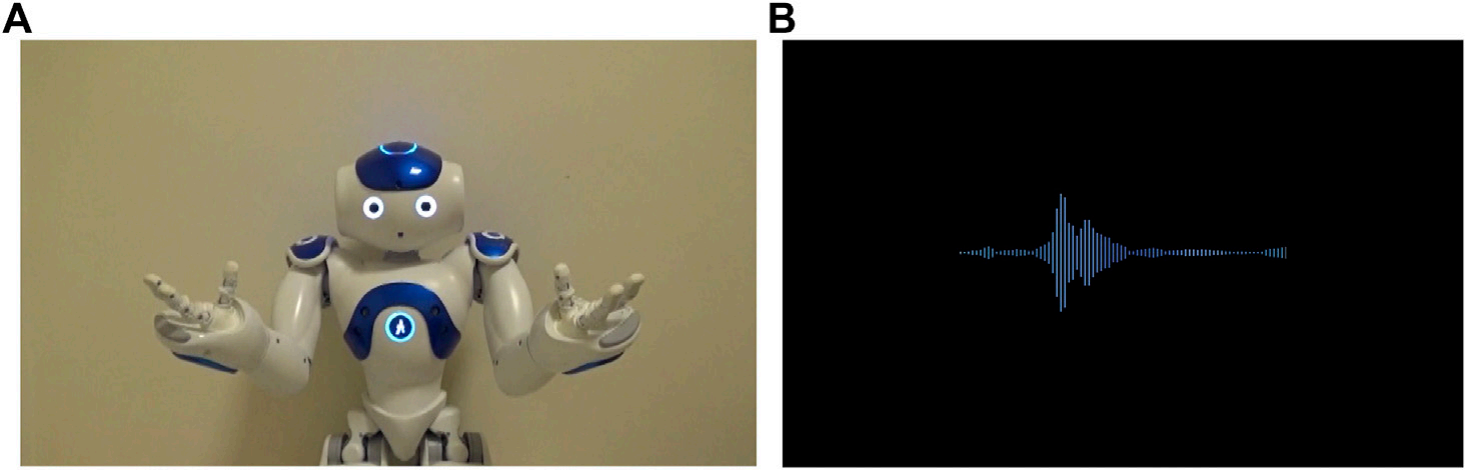
The appearance of the robot tutor **(A)** and the voice tutor **(B)**. See Supplementary Material for the videos of the robot and voice tutors).

The experiment was completed via the online video call software *Zoom* (https://zoom.us) in two sessions. In the first session, participants first filled out a demographic form. They then completed a short English language test (Quick Placement Test; UCLES, 2001), and a questionnaire assessing their attitudes toward robots, L2 anxiety, personality traits, and their impression of the robot or voice tutor. The test and questionnaires were administered using the online survey platform Qualtrics (https://www.qualtrics.com). Then, participants received a one-on-one English lesson either from the robot or the voice tutor. For the lesson, participants were sent to a breakout room^1^, and participants were alone with the tutor. Immediately after the lesson, participants in both conditions completed two measures of learning (i.e., immediate production and receptive tests). The second session took place one week later, and participants connected via Zoom again and completed the same vocabulary tests (i.e., delayed production and receptive tests). The same set of tests and surveys were administered in the robot tutor and voice tutor conditions, but in the voice tutor condition, the term “voice assistant” was used in place of “robot” for the surveys on the attitudes, impressions, and preference (see Figure 1 for a schematic representation of the procedure, and Figure 2 for the appearance of the robot and voice-only tutors).

#### Negative Attitudes Toward Robots

*Negative Attitudes toward Robots Scale* (NARS; Nomura et al., 2006) was used to assess attitudes toward robots. The NARS consists of 14 questions divided into three subordinate scales: negative attitude toward interacting with robots (S1), negative attitude toward the social influence of robots (S2), and negative attitude toward emotions involved in the interaction with robots (S3). The Turkish version of the NARS (Kanero et al., 2021) was used. Participants rated how well each of the statements represented their attitudes toward robots on a scale of 1–5 (1: I strongly disagree/Kesinlikle katılmıyorum, 2: I disagree/Katılmıyorum, 3: Undecided/Kararsızım, 4: I agree/Katılıyorum, 5: I strongly agree/Kesinlikle katılıyorum). In the voice tutor condition, the word “robot” on the NARS scale was replaced by “voice assistant.”

#### Impressions of the Robot Tutor

To assess participants’ impressions of the robot/voice tutor, we administered an impression survey with 17 questions used by Gunaydin and her colleagues (2017; available publicly at https://osf.io/nhmtw/?view_only=9f6efafeba4b48dc9b6a73b6a3d145ee). The survey shows a photograph of the robot or voice tutor, depending on the condition, and consists of two parts: The first eight questions ask participants to rate their willingness to engage and interact with the target in the future (e.g., This robot/voice assistant seems like a robot/voice assistant I would like to get to know/Tanımak istediğim bir robot/sesli asistan gibi gözüküyor) on a scale of 1–7 (1: I fully disagree/Hiç katılmıyorum, 2: I disagree/Katılmıyorum, 3: I somewhat disagree/Kısmen katılmıyorum, 4: I neither agree nor disagree/Ne katılıyorum ne de katılmıyorum, 5: I somewhat agree/Kısmen katılıyorum, 6: I agree/katılıyorum, 7: I fully agree/Tamamen katılıyorum). The next nine questions ask participants to rate how their interaction will be with the robot/voice assistant (e.g., How much do you think you will like this robot/voice assistant?/Bu robotu/sesli asistanı ne kadar seveceğinizi düşünüyorsunuz?) on a scale of 1–7 (1: Not at all/Hiç, 7: Very much/Çok fazla). After the lesson, participants rated the same items but were told to rate the statements based on their interactions with their tutor. The original survey in English was translated into Turkish by the second author and research assistants who are native speakers of Turkish. To adapt to our study, the word “person” was replaced with “robot” for the robot tutor condition and “voice assistant” for the voice tutor condition.

#### L2 Anxiety

The Turkish version of the Foreign Language Classroom Anxiety Scale (FLCAS; Horwitz et al., 1986) translated by Aydın et al. (2016) was administered. The FLCAS consists of 33 statements (e.g., I never feel quite sure of myself when I am speaking in my foreign language class/Yabancı dil derslerinde konuşurken kendimden asla emin olamıyorum.) to be rated on a scale of 1–5 (1: I fully disagree/Hiç katılmıyorum, 2: I disagree/Katılmıyorum, 3: I neither agree nor disagree/Ne katılıyorum ne de katılmıyorum, 4: I agree/Katılıyorum, 5: I fully agree/Tamamen katılıyorum).

#### Personality Traits

The Turkish version of a personality inventory was used to test the five personality traits – openness to experience, conscientiousness, extroversion, agreeableness, and neuroticism (Demir and Kumkale, 2013). This survey included 44 questions addressing each of the five traits – 7 items for conscientiousness (e.g., I stick to my plans/Yaptığım planlara sadık kalırım); 10 items for neuroticism (e.g., I am depressed/Depresifimdir); 9 items for each of openness to experience (e.g., My interests are very diverse/İlgi alanlarım çok çeşitlidir), extroversion (e.g., I am talkative/Konuşkanımdır), and agreeableness (e.g., I am helpful/Yardımseverimdir). Participants rated how well each of the statements represented their personality on a scale of 1–5 (1: I strongly disagree/Kesinlikle katılmıyorum, 2: I disagree/Katılmıyorum, 3: I neither agree nor disagree/Ne katılıyorum, ne de katılmıyorum, 4: I agree/Katılıyorum, 5: I strongly agree/Kesinlikle katılıyorum).

#### Post-Lesson Vocabulary Tests

Immediately after the lesson, we first administered the production vocabulary test (hereafter the immediate production test), and then the receptive vocabulary test (hereafter the immediate receptive test). To assess to what extent vocabulary was retained over time, participants completed the same measures again after a delay of one week (delayed post-lesson tests). The definitions of the target words used in the production test were the same as the definitions used in the lesson. In the receptive test, the pictures from the Peabody Picture Vocabulary Test, Fourth Edition (PPVT-4; Dunn and Dunn, 2007), which correspond to the target words, were used. In the production test, the experimenter provided the definitions of the learned English words one by one in a randomized order, and the participant was asked to say the corresponding English word. In the receptive test, the participant heard the learned English word and was asked to choose a picture that matched the word from four options. The delayed post-lesson tests were conducted via Zoom seven days after the lesson. Due to schedule conflicts, however, three participants in the robot tutor condition and two participants in the voice tutor condition completed these tests after six days, while four participants in each condition completed the tests after eight days. Also, three participants in the voice tutor condition completed the test after nine days.

#### Tutor Preference

After the delayed post-lesson tests, we also asked participants to rate how much they want to learn English from a human, a robot, and a voice assistant. A scale of 1–5 was used (1: I certainly do not want/Kesinlikle istemem, 2: I do not want/İstemem, 3: I neither want nor not want/Ne isterim ne istemem, 4: I want/İsterim, 5: I certainly want/Kesinlikle isterim).

#### English Lesson With the Robot or Voice Tutor

Following the previous study (Kanero et al., 2021), participants were taught eight English nouns – upholstery, barb, angler, caster, dromedary, cairn, derrick, and cupola (see Table 1; see Kanero et al. (2021) for the details of the word selection process).

**TABLE 1:** The target words and their definitions used in the study.

Word	Definition
Upholstery	*Bu kelime döşemelik kumaş anlamına gelir* (This word means fabric used to make a soft covering)
barb	*Bu kelime çengel ya da kanca anlamına gelir* (This word means the tip of an arrow or fishhook)
Angler	*Bu kelime olta ile balık tutan kimse anlamına gelir* (This word means a person who fishes with hook and line)
Caster	*Bu kelime bir şeye takılan küçük tekerlek anlamına gelir* (This word means a little wheel attached to something)
Dromedary	*Bu kelime tek hörgüçlü deve anlamına gelir* (This word means a one-humped camel)
Cairn	*Bu kelime taş yığını anlamına gelir* (This word means a mound of stones)
Derrick	*Bu kelime petrol kuyusu üzerindeki kule anlamına gelir* (This word means a tower over an oil well)
Cupola	*Bu kelime bir çatı üstüne inşa edilen küçük kubbe benzeri yapı anlamına gelir* (This word means a rounded vault-like structure built on top of a roof)

In both tutor conditions, the robot or voice tutor briefly chatted with the participant and explained the structure of the lesson first, and then introduced the words one by one. Each target word was taught in four steps:

1) The tutor introduced the target L2 (English) word and asked the participant whether the participant already knew the word (Note that none of the participants knew any of the words).
2) The tutor introduced the definition of the target word in L1 (Turkish, see Table 1).
3) The tutor asked the participant to utter the target word following the tutor, three times.
4) The tutor again defined the word and asked the participant to repeat the definition.

After learning every two target words, the participant was given a mini quiz in which the tutor provided the definitions of the target words and asked the participant for the corresponding word. The lesson lasted for about 15 min. At the end of the lesson, the robot or the voice tutor asked the participant to return to the previous room and find the experimenter they met prior to the lesson. The human experimenter administered the immediate production and receptive vocabulary tests.

To use the same voice in English and Turkish speech, we recorded the speech of a female bilingual experimenter and added sound effects to make the speech sound robot-like. The same set of speech sounds were used for both the robot and voice tutors. The visuals of both tutors were presented as a series of seamlessly transitioning videos on Zoom. The movements of the robot tutor were filmed (see Figure 2A), whereas the soundwaves of the voice tutor were created using Adobe After Effect (https://www.adobe.com/products/aftereffects.html; Figure 2B).

The robot tutor provided no facial expressions but moved its head and arms during the lesson to keep the participant engaged. Most actions were chosen from the Animated Speech library of SoftBank Robotics (http://doc.aldebaran.com/2-1/naoqi/audio/alanimatedspeech_advanced.html), although some were created by the first author to better suit the lesson.^2^ While pronouncing the target L2 word and its definition, the robot stood still without any movements to avoid the motor sound of the robot hindering the hearing. There were unavoidable behavioral differences between the two tutors (e.g., the motor sound of the robot), but otherwise, the differences between the two tutors were kept minimal.

## Results

### Robot vs. Voice Tutor

**FIGURE 3:**
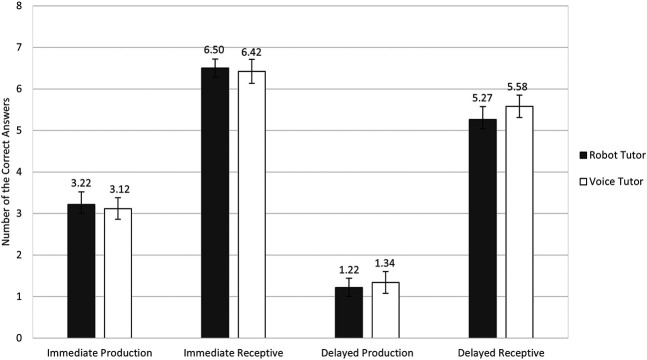
Mean number of correct answers in the robot tutor and voice tutor conditions in the four post-lesson tests. *N* = 100 for the immediate production and receptive tests; *N* = 99 for the delayed production and receptive tests. The highest possible score for each test was eight. The error bars indicate the standard errors.

We first examined if participants in the robot tutor and voice tutor conditions differed in their post-lesson test scores. We compared the two tutor conditions across all four learning outcome measures: immediate production test, immediate receptive test, delayed production test, and delayed receptive test. We conducted simple Generalized Linear Mixed Models (GLMMs) on each post-lesson test with Tutor Type (robot vs. voice) as a fixed effect and Word as random intercepts.^3^ In this model, we also added the pre-lesson English test scores as an additional fixed effect to control for the difference in English proficiency between the conditions. As shown in Figure 3, participants did not differ in terms of learning outcomes across conditions (immediate production test, *B* = 0.01, *SE* = 0.16, *Z* = 0.04, *p* = 0.968; delayed production test, *B* = -0.29, *SE* = 0.20, *Z* = -1.44, *p* = 0.149; immediate receptive test, *B* = 0.04, *SE* = 0.18, *Z* = 0.20, *p* = 0.845; or the delayed receptive test, *B* = -0.26, *SE* = 0.16, *Z* = -1.64, *p* = 0.101).

### Predicting the Learning Outcomes With Individual Difference Factors

Next, we examined whether some participants learned better or worse from robots depending on their attitudes toward robots, the first impression of the robot or voice tutor, anxiety in L2 learning, and personality traits. As indicated by Cronbach’s alphas in Table 2, each of these variables was measured reliably. Therefore, items measuring each construct were averaged to create relevant indices. For NARS, L2 anxiety, and personality, values ranged between 1 and 5. Higher values for NARS indicated having more negative attitudes toward robots; similarly, higher values for L2 Anxiety indicated having greater anxiety. For the impression survey, the values ranged between 1 and 7 and higher values indicated a more positive first impression.

**TABLE 2:** Descriptive statistics for the individual difference measures.

	Robot tutor		Voice tutor	
	*α*	*Mean*	*SD*	*Mean*	*SD*
NARS (14)	0.88	2.71	0.71	2.55	0.63
L2 anxiety (33)	0.95	2.57	0.79	2.48	0.69
Personality (44)					
Openness (9)	0.76	4.13	0.55	4.15	0.47
Conscientiousness (7)	0.81	3.20	0.74	3.22	0.59
Extroversion (9)	0.92	3.64	0.87	3.86	0.82
Agreeableness (9)	0.77	3.94	0.52	3.85	0.58
Neuroticism (10)	0.86	3.37	0.75	3.35	0.71
Impression (17)	0.92	4.19	1.27	3.51	1.02

N = 100. The number in parenthesis indicates the number of items in the scale.

### Negative Attitudes Toward Robots

We built four separate GLMMs, one for each post-lesson test (immediate production, immediate receptive, delayed production, and delayed receptive), with Word as a random intercept to examine whether negative attitudes toward robots and voice assistants predicted the number of words participants learned. As shown in Table 3, in line with the previous study (Kanero et al., 2021), negative attitudes toward robots predicted the learning outcomes in a robot-led vocabulary lesson, though only in the delayed tests. Negative attitudes did not predict learning in the voice tutor condition.

**TABLE 3:** GLMMs with NARS as the sole predictor for the four post-lesson scores.

	Robot tutor				Voice tutor			
	*B*	*SE*	*Z*	*p*	*B*	*SE*	*Z*	*p*
Immediate production	-0.08	0.15	-0.51	0.610	0.03	0.18	0.20	0.843
Immediate receptive	-0.20	0.18	-1.14	0.253	0.01	0.20	0.07	0.947
Delayed production	-0.45	0.22	-2.01	0.045	-0.03	0.21	-0.13	0.895
Delayed receptive	-0.31	0.15	-2.08	0.038	0.05	0.18	0.29	0.771

For the immediate tests, *N* = 50 in both conditions; For the delayed tests, *N* = 49 in the robot tutor condition and *N* = 50 in the voice tutor condition.

### First Impressions of the Robot

To evaluate the relation between the first impressions of the tutor and the learning outcomes, we followed the same steps and built GLMMs separately for the two tutor conditions. As shown in Table 4, there was no significant relation between learning outcomes and first impression in either condition.

**TABLE 4:** GLMMs with the first impression as the sole predictor for the four post-lesson scores.

	Robot tutor				Voice tutor			
	*B*	*SE*	*Z*	*p*	*B*	*SE*	*Z*	*p*
Immediate production	-0.06	0.09	-0.68	0.500	0.04	0.11	0.37	0.715
Immediate receptive	0.04	0.10	0.42	0.678	0.05	0.13	0.43	0.664
Delayed production	-0.03	0.11	-0.26	0.797	0.01	0.13	0.05	0.959
Delayed receptive	0.02	0.08	0.24	0.811	-0.04	0.11	-0.33	0.738

For the immediate tests, *N* = 50 in both conditions; For the delayed tests, *N* = 49 in the robot tutor condition and *N* = 50 in the voice tutor condition.

### L2 Anxiety and Personality Traits

The influence of L2 learning anxiety was similarly examined by building a GLMM for each post-lesson test for the robot tutor and voice tutor conditions with Word as a random intercept. In the robot tutor condition, L2 Anxiety predicted the scores of most tests except the immediate receptive test; in the voice tutor condition, the significance was found in the delayed production and receptive tests (Table 5).

**TABLE 5:** GLMMs with L2 Anxiety as the sole predictor for the four post-lesson scores.

	Robot tutor condition				Voice tutor condition			
	*B*	*SE*	*Z*	*p*	*B*	*SE*	*Z*	*p*
Immediate production	-0.43	0.14	-2.97	0.003	-0.16	0.16	-1.00	0.315
Immediate receptive	-0.22	0.16	-1.35	0.176	-0.15	0.18	-0.83	0.404
Delayed production	-0.42	0.20	-2.08	0.037	-1.36	0.27	-5.04	<0.001
Delayed receptive	-0.31	0.14	-2.25	0.025	-0.41	0.16	-2.53	0.012

For the immediate tests, *N* = 50 in both conditions; For the delayed tests, *N* = 49 in the robot tutor condition and *N* = 50 in the voice tutor condition.

We also built four GLMMs for each post-lesson test to evaluate the relevance of personality traits. In concert with the previous study (Kanero et al., 2021), the personality traits were not reliable predictors of the learning outcomes of the robot-led L2 lesson. In the robot tutor condition, extroversion was positively correlated with the immediate receptive test scores (*B* = 0.41, *SE* = 0.17, *Z* = 2.35, *p* = 0.019), and agreeableness was positively correlated with the delayed receptive scores (*B* = 0.65, *SE* = 0.23, *Z* = 2.77, *p* = 0.006).

### Attitudes, Impressions, and Preferences

#### Attitudes Toward Robots

**FIGURE 4:**
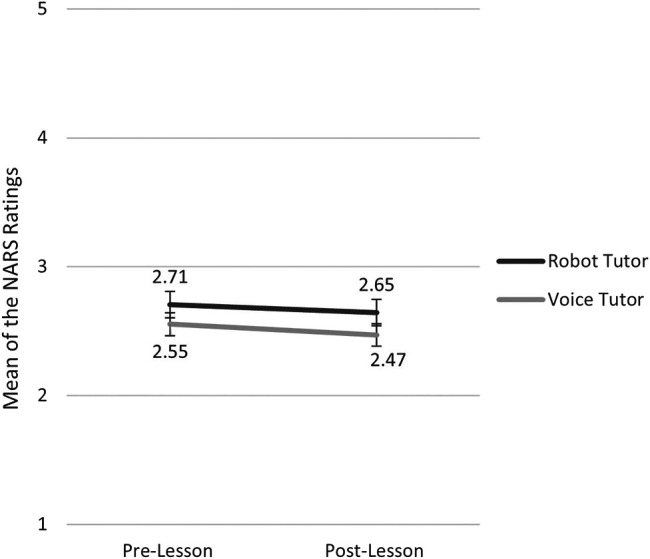
Mean of the NARS ratings in the robot tutor and voice tutor conditions before and after the lesson. *N* = 100 for the pre-lesson NARS; *N* = 99 for the post-lesson NARS. The highest possible score for each test was 5. The error bars indicate the standard errors.

With the purpose of assessing the change in attitudes after the interaction with the robot or voice tutor, the normality assumption of the data was first examined. In comparing the attitude scores between the two tutor conditions or between the pre- and post-lesson surveys, we performed a Shapiro-Wilk’s test of normality. We then used *t*-tests when the two compared data are both normally distributed, and Wilcoxon Signed-Ranks Tests when the normality assumption was violated. The difference between the tutor conditions was not significant in either before (*Z* = 0.97, *p* = 0.334) nor after the lesson [*t* (97) = 1.17, *p* = 0.244]. Negative attitudes toward robots/voice assistants did not change before and after the lesson in the robot tutor condition (*Z* = 1.10, *p* = 0.267), nor the voice tutor condition [*t* (49) = 1.65, *p* = 0.105]. In other words, interacting with the tutor did not improve learners’ attitudes toward the specific tutor (Figure 4).

#### Impressions of the Robot Tutor

**FIGURE 5:**
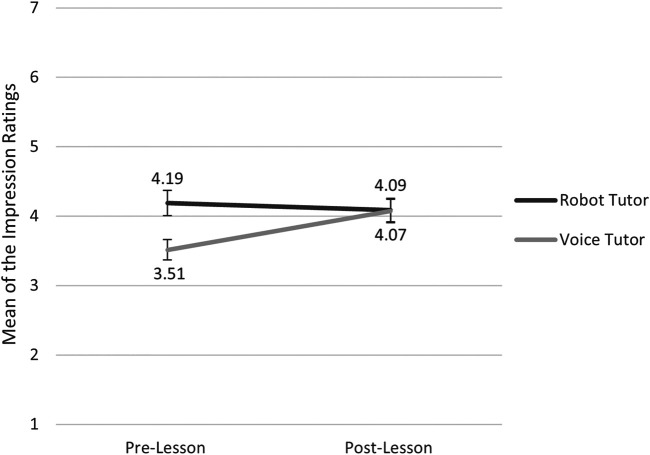
Mean ratings of the impression survey in the robot tutor and voice tutor conditions before and after the lesson. *N* = 100 for pre-lesson and *N* = 99 for post-lesson. The highest possible score for each test was 7. The error bars indicate the standard errors.

A paired sample *t*-test on the impression survey indicated that, in the robot condition, participants’ impressions of the robot before and after the lesson did not significantly change [*t* (48) = -0.22, *p* = 0.407]. In the voice tutor condition, on the other hand, the ratings were significantly higher after than before the lesson [*t* (49) = -3.78, *p* < 0.001]. In addition, independent paired *t*-tests demonstrated, that whereas the difference in the pre-lesson impression scores between the two tutor conditions was significant [*t* (98) = 2.89, *p* = 0.005], the two did not differ significantly in the post-lesson impression scores [*t* (97) = -0.06, *p* = 0.954]. These results indicate that, although the expectation was different for the two tutors, the impressions became comparable after having an actual interaction (see Figure 5).

### Preference of Tutors

**FIGURE 6:**
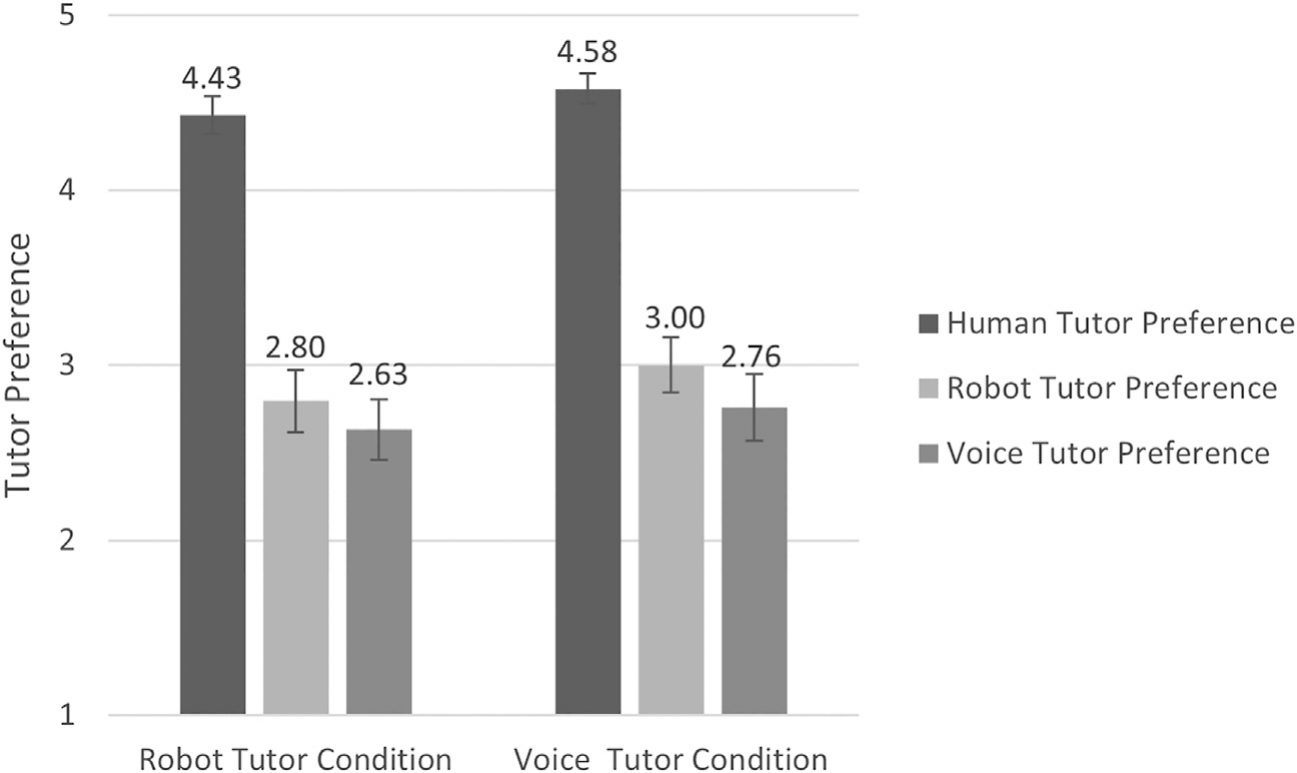
Preference ratings for each tutor after the lesson. *N* = 49 in the robot tutor condition; *N* = 50 in the voice tutor condition. The highest possible score for each test was 5. The error bars indicate the standard errors.

Wilcoxon Signed-Ranks tests suggest that participants in the robot tutor condition preferred a human tutor to a robot (*Z* = 5.30, *p* < 0.001) or a voice tutor (*Z* = 5.52, *p* < 0.001), but did not differ in their preference for a robot tutor and a voice tutor (*Z* = 1.07, *p* = 0.286; see Figure 6). In the voice tutor condition, participants also preferred a human tutor to a robot tutor (*Z* = 5.59, *p* < 0.001), and to a voice tutor (*Z* = 5.39, *p* < 0.001); and they also preferred a robot tutor to a voice tutor (*Z* = 2.15, *p* = 0.031). Participants in both tutor conditions did not significantly differ in their preference for human tutor (*Z* = -1.27, *p* = 0.206), robot tutor (*Z* = -0.85, *p* = 0.397) or voice tutor (*Z* = -0.28, *p* = 0.778).

## Discussion

As the presence of social robots in our lives is becoming more and more prominent, it is critical to understand when and for whom robots can provide the most benefit. The present study examined the physical embodiment of robots and individual differences among learners to evaluate the effectiveness of robot tutors in an online L2 vocabulary lesson. To further understand the circumstances in which the robot tutor is effective, we also assessed how learners’ individual differences in attitudes toward robots, impressions of the robot tutor, anxiety about L2 learning, and personality traits were related to their learning outcomes. Through a stringent evaluation using two different outcome measures at two time points, we found that embodiment did not affect learning in our lesson, and individuals with negative attitudes toward robots and L2 learning anxiety learned fewer words in the robot-led lesson.

### Embodiment of the Robot Tutor

The learning outcomes were comparable on all four measures between the robot tutor and voice tutor conditions, and thus we did not see an advantage of the robot tutor having a body. Our results are in concert with previous research that did not find benefits of physical embodiment in learning (e.g., Kennedy et al., 2015). To further confirm the conclusion, we also conducted an exploratory analysis comparing the current data with the data from the in-person robot lesson in our previous study (Kanero et al., 2021). We built four GLMMs for each post-lesson test examining the main effects of 1) embodiment (in-person and Zoom robot tutors vs. Zoom voice tutor), and 2) physical presence (in-person robot tutor vs. Zoom robot and voice tutors). Neither embodiment nor physical presence was identified as a significant predictor (all *p*’s > 0.080). Therefore, we found no evidence of the robot’s embodiment or physical presence affecting the learning outcomes of the simple L2 vocabulary lesson. As discussed further in *Changes in Attitudes, Impressions, and Preferences From Before to After the Lesson* section, we did not find an impact of physical embodiment on the learning outcomes or impressions of the robot tutor after the lesson either. The context of our paradigm must be taken into consideration in interpreting these results, as our vocabulary learning task was solely conversational and did not require the robot to interact with the physical world. Embodiment may not be a factor in such non-physical settings (Ligthart and Truong, 2015), hence learning environments with physical materials may yield different results.

### Individual Differences and Learning Outcomes

In concert with the previous study concerning in-person lessons (Kanero et al., 2021), we found that negative attitudes toward robots as well as anxiety about learning L2 are related to L2 vocabulary learning with a robot, though the relations were less pronounced. In addition to the measures used previously, we tested the effect of the learner’s first impression of the robot tutor. The current study was among the first to test whether 1) the first impressions of the robot affect the learning outcomes, and 2) the impressions of the robot change before and after the interaction. Contrary to our expectation, the first impression ratings did not predict the number of words participants learned from the lesson. Therefore, we found that the NARS, which assessed participants’ general attitudes toward robots, was a better predictor of the learning outcomes than the impression of the specific robot tutor.

The inclusion of the impression survey is also relevant for the discussion of the construct validity of the questionnaires used in HRI studies. Many studies used the NARS (Nomura et al., 2006) to measure the attitudes of participants and to predict participants’ behaviors (Nomura et al., 2006; Takayama and Pantofaru, 2009; Ivaldi et al., 2017). In both the current study and the previous study (Kanero et al., 2021), although the NARS predicted the number of words participants learned, the correlation was weak to moderate. One possibility was that the difference in generality between the independent variables (i.e., general attitudes toward all robots) and dependent variables (i.e., the number of words learned from a specific robot) led to the relatively weak correlations. Importantly, the impression survey in the current study was a less general measure, but we did not find a correlation between the impression and learning outcomes.

### Changes in Attitudes, Impressions, and Preferences From Before to After the Lesson

On average, participants’ attitudes toward robots (and voice assistants) became more positive after they interacted with the specific tutor, but the change was not statistically significant. It should be noted that our lesson was very short and the interaction was minimal, and we may expect a greater change when the lesson is longer and more interactive. The NARS was also tested in the previous study (Kanero et al., 2021), and thus we can compare the data of the current study with the data from the in-person lesson. We found that the learner’s negative attitudes toward robots did not significantly change before and after the in-person lesson either [*t* (49) = -1.02, *p* = 0.31]. As per participants’ impressions of the tutor, the first impressions were better for the robot tutor than the voice tutor, but the impressions became comparable between the two conditions after the actual interaction. The results may indicate that, although the impression before the lesson can be affected by embodiment, the short Zoom session was enough for the learners to override the first impressions and assess the agent based on the actual interactive and communicative capabilities. With regard to the learner’s preference, we observed a clear preference for a human tutor over both of the machine tutors, and some preference for the robot tutor over the voice tutor. These results also emphasize the importance of choosing different scales depending on what the researcher plans to evaluate.

### Limitations and Future Directions

In the current study, embodiment did not facilitate vocabulary learning, and the learner’s attitudes toward robots and anxiety about learning L2 consistently predicted learning outcomes. In terms of physical presence, however, we could only compare the current study with the previous study (Kanero et al., 2021) to anecdotally discuss its lack of impact. Therefore, a direct comparison between in-person and virtual lessons should be made before drawing a conclusion. It would also be critical to further test the unique features of robots (e.g., the ability to perform gestures) and to consider other aspects of language such as grammar and speaking (Kanero et al., 2021). Similarly, the lesson scenarios, the demographic characteristics of participants (e.g., education, familiarity with robots) and the morphology of robots (e.g., Pepper, Kismet, Leonardo) might affect learning outcomes. Future research should not only investigate the influence of these factors on learning outcomes, but also analyze the detailed nature of human-robot interaction (e.g., the learner’s behaviors during the lesson).

Perhaps most importantly, in the current study, the human-robot interaction was limited to one session lasting only about 15 min. Needless to say, more research is needed to examine whether the physical body of a robot affects learning outcomes in other settings such as a lesson on another subject, or in a longer and more interactive lesson. The effects of embodiment may be more pronounced when multiple lessons are provided over a longer period of time. Further, some researchers suggest that robot tutors may reduce the L2 anxiety of child learners in the long run (Alemi et al., 2015), and thus future research may focus on the long-term effects of robot language lessons on the anxiety levels of children and adults. Another recent study also found that children between 5 and 6 years old do not interact with voice assistants as much as they interact with humans (Aeschlimann et al., 2020). To our knowledge, no child study has compared robots and voice assistants. Overall, developmental research should adopt an experimental design similar to our study and examine whether the current findings can be replicated with a younger population.

Our data in the voice tutor condition also provide insights into the effectiveness of voice assistants such as Amazon Alexa and Apple Siri. Research with children suggests that voice assistants are perceived as a source of information that can answer questions about a wide range of subjects, including language such as definitions, spellings, and translations (Lovato et al., 2019). Our results show that adults can learn a second language from voice assistants as well, at least to the same extent they do with social robots. It should also be noted that one reason why we did not find a link between negative attitudes toward voice assistants and learning outcomes might be that we adapted a questionnaire about robots, simply by changing the word “robot” to “voice assistant.” While this manipulation made the two conditions as comparable as possible, the validity of the voice assistant questionnaire should be carefully considered. Future research may use our findings as a base to explore how and for whom voice tutors are beneficial.

Finally, we should also point out that the current study was conducted amid the COVID-19 pandemic. We believe that our findings are generalizable, and if anything, the pandemic might have provided a better setting to evaluate the impact of (dis)embodiment. Online education has become abundant, and people may be less hesitant to engage in virtual interactions, hence the difference between in-person and online interactions should be less driven by the unfamiliarity of online interactions in the current climate. Nevertheless, more studies should be conducted to critically assess the generalizability of the findings.

### Conclusion

This study was the first to empirically investigate the influence of the robot’s physical embodiment on second language learning. The study presents an example of embodiment not affecting the learning outcomes although the results should be interpreted cautiously until the results are replicated for different language learning tasks and using various scenarios and interaction designs. Evaluating the influences of individual differences in robot-led Zoom lessons, we also found that the learner’s general attitudes toward robots predict learning outcomes. Our findings provide some hope for the difficult situation during the COVID-19 pandemic because participants successfully learned vocabulary in a short Zoom lesson. The current results also encourage more researchers to be engaged in studying the influence of the user’s individual differences in human-robot interaction and policymakers and educators to carefully consider how social robots and other technological devices should be incorporated in educational settings.

## Data Availability

The datasets generated and analyzed for this study will be available from the corresponding author on reasonable request.

## Footnotes

1 Breakout room is a feature in Zoom that allows the host to split one Zoom session into multiple separate subsessions whereby participants in separate breakout rooms do not see each other. We put the participant into a separate breakout room away from the Experimenter and the tutor so that participants do not need to feel watched or pressured.

2 The gestures used in the lesson were mostly generic except that, when the participant repeated the target word following the robot tutor, the robot made the “pinched fingers” gesture where all fingers were put together with the palm side up and the hand was moved up and down. This conventional gesture means “very good” in the Turkish culture.

3 We used GLMMs in these analyses, because our data are not normally distributed, and because they allow us to analyze the responses of participants without averaging across trials (Jaeger, 2008). As the outcome (the scores of the four post-lesson tests) was a binary variable (correct vs. incorrect), logit (log-odds) was used as the link function. GLMMs were generated in R (R Development Core Team, 2016) using the *lme4.glmer* function (Bates, 2015). In all models, we included the random effect of item (e.g., L2 words) as some L2 vocabulary words may be inherently more difficult to learn than others. All models were fit by maximum likelihood using adaptive Gauss-Hermite quadrature (nAGQ = 1).

## Supplementary Material

Video 1.MP4 An excerpt from the robot tutor lesson.

Video 2.MP4 An excerpt from the voice tutor lesson.
